# Correlation of single nucleotide polymorphisms in the pregnancy-associated plasma protein-A gene with carotid plaques

**DOI:** 10.1186/s12872-015-0041-1

**Published:** 2015-06-30

**Authors:** Shiming Zhou, Min Cui, Zegang Yin, Rui Li, Jie Zhu, Huadong Zhou

**Affiliations:** Department of Neurology, Daping Hospital, Third Military Medical University, No. 10 Changjiang Branch Road, Daping, Chongqing, 400042 China

**Keywords:** Calcified plaque, Carotid plaque, Pregnancy-associated plasma protein A, Single nucleotide polymorphisms

## Abstract

**Background:**

Pregnancy-associated plasma protein A (PAPP-A) is abundantly expressed in carotid plaques. This study investigated the association between single nucleotide polymorphisms (SNPs) of *PAPP-A* and the presence of carotid plaques.

**Methods:**

A total of 408 patients with carotid plaques and 493 controls were included in the study. All subjects were Southern Chinese Han. Carotid plaques were analyzed by computer tomography angiography. *PAPP-A* SNPs were identified by ligase detection reaction-polymerase chain reaction analysis. The *PAPP-A* genotypes rs3747823, rs7020782, and rs13290387 were analyzed.

**Results:**

The rs7020782 C allele genotype correlated with an increased risk of developing carotid plaques under the dominant, recessive, and additive models (adjusted odds ratios: 2.60, 2.36, and 3.48, respectively; *P* ≤ 0.001). Only C allele-carrying genotypes correlated with a significantly increased risk of carotid plaque based on studies stratified by age and sex under the dominant model. rs7020782 remained significantly associated with the risk of carotid plaque calcification after adjusting for age and potential confounders (adjusted odds ratio, 1.89; 95 % confidence interval, 1.17–3.08; *P* = 0.010).

**Conclusions:**

This study found, for the first time, that the A˃C variation of rs7020782 might be an independent risk factor for carotid plaque development and calcification. The determination of such genotypes could provide a new tool for identifying individuals at high risk for carotid atherosclerosis.

**Electronic supplementary material:**

The online version of this article (doi:10.1186/s12872-015-0041-1) contains supplementary material, which is available to authorized users.

## Background

Pregnancy-associated plasma protein A (PAPP-A) is a metalloproteinase that regulates insulin-like growth factor (IGF) and acts as a biomarker of inflammation [1–4]. It is abundantly expressed in carotid plaques, which form an important pathological basis of strokes. A number of studies have reported that its increased levels might be recognized as a promising biomarker for acute coronary syndromes [5–7], cerebrovascular events [8, 9], and systemic atherosclerotic disease [10]. It has also been suggested that PAPP-A concentrations could be a marker for carotid atherosclerosis [11] as well as carotid plaque destabilization and rupture [12, 13].

Although PAPP-A appears to be a key factor in the progressive advanced stage of atherosclerosis [14], its precise role outside pregnancy is yet to be clarified [15]. *PAPP-A* expression has been linked with carotid vulnerable plaque development, and serum PAPP-A levels with carotid atherosclerosis. Therefore, the association between PAPP-A and carotid plaques deserves further research [13, 16].

A previous study revealed an association between *PAPP-A* polymorphisms and acute myocardial infarction [17]. Additionally, in 2012, Wang et al. reported that genetic polymorphisms of *PPAP-A* were correlated with ischemic stroke [18]. More recently, another study investigated the relationship between serum PAPP-A levels and coronary plaque characteristics [19]. However, little is known about the association between *PAPP-A* polymorphisms and carotid plaques.

China has a growing population that is increasingly aging, and has seen a rapid rise in the incidence of cerebrovascular events. The prevention and treatment of these is therefore an important public health problem. The identification of patients at high risk of developing carotid plaques may help the prevention of cerebrovascular events. Therefore, in our present study, we selected three single nucleotide polymorphisms (SNPs) of *PAPP-A* (rs3747823, rs7020782 and rs13290387) according to literature [18, 17] may be related to vascular diseases to investigate the association of *PAPP-A* polymorphisms with carotid plaque development.

## Methods

### Subjects

The study included a total of 901 subjects aged 50 years and above; 408 subjects had carotid plaques and 493 were controls without carotid plaques. Those subjects diagnosed with carotid atherosclerosis by computed tomography angiography (CTA) were enrolled at the Division of Neurology of Daping Hospital (Chongqing, China) between January 2011 and December 2012. The controls were selected during the same period from the same hospital, and were shown not to have carotid atherosclerosis by CTA. All subjects were Southern Chinese Han. CTA carotid plaque characterization was defined as follows: (1) cut-off value to differentiate lipid core from fibrous tissue and fibrous tissue from calcifications: 60 Hounsfield Units (HU) and 130 HU, respectively [20], based on multi-detector CTA measurements; (2) cut-off point between calcifications and non-calcified tissue: 130 HU [20]; (3) cut-off point between lumen and atherosclerotic vessel wall: 200 HU [20]. Two experienced radiologists and two neurologists who were both blinded to the genotype read the scans and reached an agreement about diagnosis. The Ethics Committee of Daping Hospital approved the study and all subjects signed an informed consent form.

Exclusion criteria included patients with: (1) acute coronary syndrome; (2) liver or kidney dysfunctions or severe heart failure; (3) infections or immune system diseases; (4) peripheral vascular diseases; (5) cerebral infarction induced by arteritis, blood diseases, cancer, drugs, aneurysms, or vascular malformations.

### Collection of clinical data and laboratory measurements

Subject body weight and height were measured directly. Blood pressure was measured with a mercury-column sphygmomanometer after 10 min of rest in the supine position. Body mass index (BMI) was calculated using body weight (kg) divided by squared height (m^2^). Fasting plasma glucose (FPG), total plasma cholesterol (TC), triglycerides (TG), low density lipoprotein cholesterol (LDL-C), and high density lipoprotein cholesterol (HDL-C) were measured using standard enzymatic techniques.

Blood samples (10 ml) were drawn in the morning after an overnight fast from the peripheral vein of supine patients. The blood sample was immediately separated by centrifugation at 2,000 × *g* for 15 min at 4 °C. After centrifugation, the separated plasma samples were frozen at −80 °C until analysis. The leukocytes were collected in Eppendorf tube for genetic analysis. Smoking was defined as regular cigarette smoking or the use of any tobacco products on a weekly basis or more often, or regular smoking in the past [21].

### Genotyping

Genomic DNA was extracted from peripheral blood leukocytes using a commercial blood DNA extract kit (Axygen AxyPrep ™ Mag Blood Genomic DNA Kits, USA) and was stored at −20 °C until used for genotype testing. Genotype data of the Chinese Han sample (CHB sample) were downloaded from the HapMap database (http://www.hapmap.org, HapMap Public Release #2), and the minor allele frequencies of the three *SNPs* > 0.05. The genotyping of rs3747823, rs7020782 and rs13290387 was carried out by the Shanghai BioWing Applied Biotechnology Company (http://www.biowing.com.cn/) using ligase detection reaction-polymerase chain reaction (LDR-PCR) [22–26]. The primer and probe sequences and PCR and LDR product lengths of the variant are summarized in Additional file 1: Table S1. Fragment amplification was carried out in 20 μl of multiplex PCR mixture containing 50 ng (1 μl) of genomic DNA, 2 μl of 1 × buffer, 0.6 μl of Mg ^2+^(3 mM), 2 μl of dNTPs (2 mM), 0.3 μl of Taq polymerase (1U), 4 μl of 1 × Q-solution, 0.4 μl of primer mix and 9.7 μl of ddH_2_O. The PCR included initial denaturing at 95 °C for 15 min, followed by 35 cycles of denaturing at 94 °C for 30 s, annealing at 56 °C for 30 s, and extension at 72 °C for 1 min. The reaction was completed by a final extension at 72 °C for 10 min. Reactions were performed on a thermal cycler Gene Amp PCR system 9600 (Perkin Elmer, Waltham, MA, USA). Further amplification was performed in a 10 μl volume of multiplex LDR reaction mixture, containing 1 μl (100 ng) of the resultant probe mix, 1 μl of probe mix (12.5pmol/μl), 0.05 μl NEB Taq DNA ligase (2U) and 7.95 μl of ddH_2_O. The LDR conditions included initial denaturing at 95 °C for 2 min, followed by 30 cycles of denaturing at 94 °C for 15 s and annealing at 50 °C for 25 s. LDR products (1 μl) were mixed with 1 μl of ROX (ABI, Foster City, CA, USA) and 1 μl of loading buffer, detected in an ABI PRISM 377 DNA Sequencer, and analyzed with Gene-mapper (ABI, Foster City, CA, USA).

### Statistical analysis

Statistical analysis was performed using the PASW statistics 18 (International Business Machines Corp., New York, USA). Categorical variables were compared using the chi-squared test (*χ*^2^), and continuous variables compared using the Student’s *t*-test. Hardy–Weinberg equilibrium (HWE) was assessed by the *χ*^2^-test. Distributions of genotypes between cases and controls were also analyzed by the *χ*^2^-test. To assess the association of *PAPP-A* SNPs with carotid plaques, univariate and multivariate logistic regression analysis were used to calculate crude and adjusted odds ratios (ORs) with 95 % confidence intervals (95 % CI). Confounding risk factors such as age, sex, BMI, smoking, blood pressure, FPG, TC, TG, LDL-C, and HDL-C were assessed for significance using the logistic regression model. A two-tailed test of *P* < 0.05 was considered statistically significant.

## Results

The clinical characteristics of the subjects are shown in Table 1. Compared with controls, subjects with carotid plaques were significantly older, with a significantly higher incidence of males, and significantly higher systolic blood pressure, higher diastolic blood pressure, and lower HDL-C levels (*P* < 0.05). There were no significant differences in FPG, TC, TG, LDL-C, BMI, or smoking between the two groups.

**Table 1:** Clinic characteristics of subjects with carotid plaque and controls

Characteristics	Carotid plaque (*n* = 408)	Controls (*n* = 493)	*P* value
Age (years)	68.29 ± 9.80	64.89 ± 9.16	<0.001
Female (%)	44.36	57.4	<0.001
BMI (Kg/m^2^)	23.66 ± 3.98	23.60 ± 3.21	NS
Systolic blood pressure (mmHg)	136.35 ± 16.18	127.75 ± 16.48	<0.001
Diastolic blood pressure (mmHg)	81.49 ± 10.21	76.02 ± 9.47	<0.001
Fasting plasma glucose (mmol/L)	5.53 ± 1.81	5.60 ± 1.68	NS
Smoking (%)	33.58	30.43	NS
TC (mmol/L)	4.75 ± 1.05	4.85 ± 1.11	NS
TG (mmol/L)	1.54 ± 1.11	1.50 ± 1.11	NS
LDL-C (mmol/L)	2.58 ± 0.74	2.59 ± 0.75	NS
HDL-C (mmol/L)	1.14 ± 0.31	1.22 ± 0.33	<0.001

Value expressed by mean ± SD

BMI, body mass index; TC, total cholesterol; TG, triglyceride; LDL-C, low density lipoprotein cholesterol; HDL-C, high density lipoprotein cholesterol; NS, no significance

*PAPP-A* SNP genotypes in carotid plaque and control groups are shown in Table 2. The genotype distributions of all SNPs were compatible with HWE in both the carotid plaque and control groups (*P* > 0.05, omitted). There was no significant difference in the distributions of rs3747823 or rs13290387 genotypes between the carotid plaque and control groups under the three models. However, there was a significant difference in the distribution of the rs7020782 genotype under all three models between the carotid plaque and control groups (*P* < 0.001).

**Table 2:** Association study of four SNPs in PAPP-A under different models in two groups

	Carotid plaque		Controls		
Analyzing Model: SNPs	n^1^	Frequency (%)	n^1^	Frequency (%)	*P* value ^§^
rs3747823					
Additive: GG/AG/AA	263/126/18	64.6/31.0/4.4	298/155/27	62.1/32.3/5.6	NS
Dominant: (AA + AG)/GG	144/263	35.4/64.6	182/298	38.0/62.0	NS
Recessive: AA/(AG + GG)	18/389	4.4/95.6	27/453	5.6/94.4	NS
rs7020782					
Additive: AA/AC/CC	166/190/51	40.8/46.7/12.5	302/159/29	61.6/32.4/6.0	<0.001
Dominant: (AC + CC)/AA	241/166	59.2/40.8	188/302	38.4/61.6	<0.001
recessive: CC/(AC + AA)	51/356	12.5/87.5	29/461	5.9/94.1	0.001
rs13290387					
Additive: GG/CG/CC	207/163/31	51.6/40.6/7.7	269/179/35	55.7/37.1/7.2	NS
Dominant: (CG + CC)/GG	194/207	48.4/51.6	214/269	44.3/55.7	NS
Recessive: CC/(CG + GG)	31/370	7.7/92.3	35/448	7.2/92.8	NS

^§^ *P*-values were calculated by Pearson Chi-Squared test. NS, no significance

^1^The number did not add up to 408 or 493, because the SNPs genotyping with LDR method had the absent value inevitably

As shown in Table 3, logistic regression analysis revealed that the rs7020782 genotype was associated with an increased risk of carotid plaques in dominant, recessive, and additive models. After adjusting for age, gender, systolic and diastolic blood pressure, and HDL-C, a significant correlation still remained (adjusted OR, 2.60, 2.36, 3.48, respectively; 95 % CI, 1.93–3.48, 1.42–3.92, 2.05–5.92, respectively; *P* < 0.001).

**Table 3:** Risk of carotid plaque under different models of PAPP-A SNPs

SNPs analyzing Model^‡^	Crude OR (95 % CI) ^1^	*P* value^1^	Adjusted OR (95 % CI)^§^	*P* value^§^
rs7020782				
Dominant	2.33 (1.79-3.05)	<0.001	2.60 (1.93-3.48)	<0.001
Recessive	2.28 (1.41-3.66)	0.001	2.36 (1.42-3.92)	0.001
Additive	3.20 (1.95-5.24)^†^	<0.001	3.48 (2.05-5.92)^†^	<0.001

^1^ Un-adjusted logistic regression model; OR, odds ratio

^§^Adjusted for age, sex, systolic blood pressure, diastolic blood pressure, high density lipoprotein cholesterol

^‡^In analyses, a additive effect of the variant (V) allele was assumed, the genotype wild (W) W/W,W/V and V/V were coded as 1,2, and 3, respectively; when a dominant effect was assumed, genotype W/W was coded as 2, and an W/V and V/V combined were coded as 1. Accordingly, scores of 2 for W/W, W/V combined and of 1 for V/V were used in the assumed recessive effect

^†^Referred to AC vs. AA; CC vs. AA was not significant in additive model in logistic regression

**Fig. 1:**
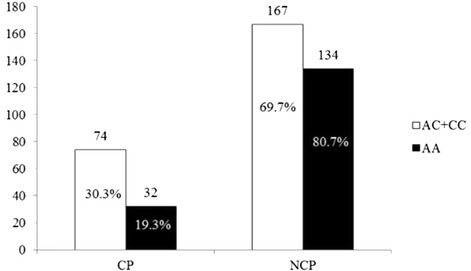
Dominant model of rs7020782 C allele in subjects with calcified plaque and non-calcified plaque. Chi-Squared test for categorical values Pearson *χ* ^2^ = 6.665, *P* = 0.010 for genotype frequency. CP: calcified plaque NCP: non-calcified plaque. Adjusted for age, sex, systolic blood pressure, diastolic blood pressure, high density lipoprotein cholesterol, the risk of calcified plaque: odds ratio, 1.89, 95 % CI,1.17-3.08, *P* = 0.010

The significant association of *PAPP-A* SNPs with plaque characteristics in the patient group is shown in Fig. 1. The rs7020782 genotype correlated with calcified and non-calcified plaque under the C allele dominant model. The *χ*^2^-test revealed that the C allele dominant model of rs7020782 was associated with an increased risk of carotid calcified plaque (OR, 1.86; 95 % CI, 1.16–2.98; *P* = 0.010). After adjusting for age and other potential confounders by multivariate logistic regression analysis, a significant correlation of rs7020782 with an increased risk of carotid calcified plaque still remained (OR, 1.89; 95 % CI, 1.17–3.08, *P* = 0.010). This indicates that the rs7020782 genotype is associated with a risk of calcified plaque in the C allele dominant model. C allele variations showed no association with plaque calcification in recessive or additive models. Moreover, the other two SNPs did not appear to be associated with carotid plaque calcification under all three models.

**Fig. 2:**
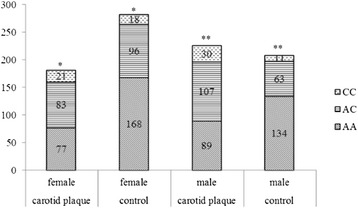
Stratified distribution of rs7020782 genotypes by sex in subjects with carotid plaque and controls. Adjusted for age, systolic blood pressure, diastolic blood pressure, high density lipoprotein cholesterol, the risk of carotid plaque under C allele dominant model in female subjects*: odds ratio, 2.08; 95 % CI, 1.38–3.14; *P* < 0.001, male subjects** : odds ratio, 3.14; 95 % CI, 2.05–4.81; *P* < 0.001

**Fig. 3:**
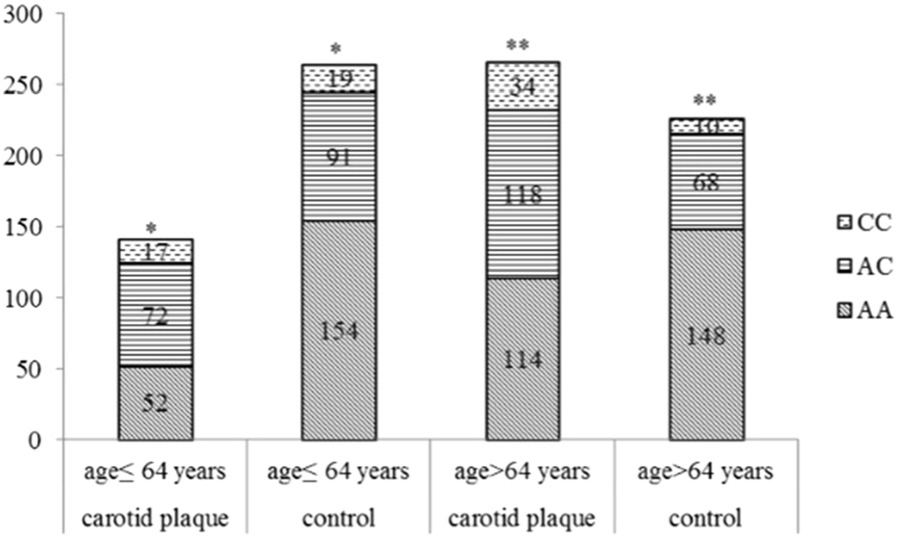
Stratified distribution of rs7020782 genotypes by age in subjects with carotid plaque and controls. Adjusted for sex, systolic blood pressure, diastolic blood pressure, high density lipoprotein cholesterol, the risk of carotid plaque under C allele dominant model in subjects aged ≤64 years *: odds ratio, 2.33; 95 % CI, 1.47–3.69; *P* < 0.001, those aged > 64 years** : odds ratio, 2.57; 95 % CI, 1.75–3.76; *P* < 0.001

We also observed a remarkable mutual effect between C allele-carrying genotypes and an increased risk of carotid plaque based on stratified analyses by sex and age under the dominant model (Figs. 2 and 3). After adjusting the variables of confounding factors, an elevated risk of carotid plaque correlated with the C allele dominant model was evident in female subjects (OR, 2.08; 95 % CI, 1.38–3.14; *P* < 0.001), male subjects (OR, 3.14; 95 % CI, 2.05–4.81; *P* < 0.001), subjects aged ≤64 years (OR, 2.33; 95 % CI, 1.47–3.69; *P* < 0.001), and those aged > 64 years (OR, 2.57; 95 % CI, 1.75–3.76; *P* < 0.001). Under the recessive model, no significant correlation was found in female subjects or those aged ≤64 years. Under the additive model, no significant correlation was found in subjects aged ≤64 years. These results indicate that only the C allele dominant model variant was significantly associated with carotid plaque development in each stratified study.

## Discussion

The present study investigated whether *PAPP-A* polymorphisms contributed to susceptibility to carotid plaques in a Southern Chinese Han population containing 408 patients and 493 controls. Our results showed that the SNP rs7020782 was significantly associated with an increased risk of carotid plaques, but no association was found for rs3747823 or rs13290387.

PAPP-A belongs to the metzincin superfamily of metalloproteinases [2] and is the founding member of a new metzincin subfamily, which differs from the four existing subfamilies (matrix metalloproteases, astacins, adamalysins/reprolysins, and serralysins) [15]. Djurić et al. [27] found that the *MMP-1* − 1062 G/2G polymorphism and specific haplotypes of three other promoter polymorphisms were significantly and independently associated with the occurrence of carotid plaques, while Li et al. [28] reported that the inter-individual variability in *MMP-12* variation may not be a risk factor for carotid plaques in the Chinese Han population.

Dong et al. [29] conducted a follow-up association study of linkage regions, which identified multiple candidate genes for carotid plaque development in a population from the Dominican Republic. In a family study, evidence for association was found regarding several genes (*NAV2*, *EFCAB11/TDP1*, *AGBL1*, *PTPN9*, *LINGO1*, and *LOC730118*), with the strongest association at rs4143999 near *EFCAB11/TDP1*. Osteoprotegerin gene (*TNFRSF11B*) polymorphisms were also recently shown to be potential markers for carotid calcified plaques in patients with carotid atherosclerosis [30]. The observed coronary artery calcification was associated with the human chromosome 9p21 locus SNPs [31], which prompted the research of genetic variants with vascular calcification and the underlying mechanism of vascular diseases [32–35].

*PAPP-A* has previously been investigated as a candidate gene for cerebrovascular diseases. A retrospective study also showed that C allele-carrying genotypes of rs7020782 have a higher risk of developing ischemic stroke compared to the GG genotype among a Northern Chinese Han population [18]. Thus far, however, few studies have explored the relationship between *PAPP-A* variants and carotid plaques, although the present study identified an association with SNP rs7020782. This SNP is in exon 14 of *PAPP-A*, and was previously reported to be associated with recurrent pregnancy loss [36]. The A > C allele variant (Tyr/Ser) of rs7020782 may influence PAPP-A protein determination in risk assessment screening during the first trimester of pregnancy [37].

Although the development of carotid plaques appears to be associated with *PAPP-A* SNP rs7020782, the precise molecular mechanism behind this development remains unclear [38]. Our study was limited by including only a relatively small number of subjects and investigating only three SNPs, so future studies of larger sample sizes and more *PAPP-A* SNPs should be carried out. The association between PAPP-A levels and *PAPP-A* polymorphisms rs7020782 should therefore be evaluated in future work to illuminate whether this SNP in exon 14 of *PAPP-A* influences serum PAPP-A concentrations.

## Conclusions

In summary, to our knowledge this is the first study to investigate the association of *PAPP-A* SNPs with the risk of carotid plaque in a Chinese Han population. Our results indicate that SNP rs7020782 contributes to carotid plaque susceptibility in this cohort.

## Additional file

Additional file 1: Table S1. Primer and probe sequence and PCR and LDR product length of PAPP-A variants.
