# Supplementary material for: Correlation of single nucleotide polymorphisms in the pregnancy-associated plasma protein-A gene with carotid plaques
Source: BMC Cardiovasc Disord. 2015 Jun 30;15:60. doi: 10.1186/s12872-015-0041-1 (PMC4485363; doi:10.1186/s12872-015-0041-1)
Supplement: Additional file 1: Table S1. — Primer and probe sequence and PCR and LDR product length of PAPP-A variants. [file 12872_2015_41_MOESM1_ESM.pdf]

Additional table S1 Primer and probe sequence and PCR and LDR product length of *PAPP-A* variants

Variant	Primer or Probe	Sequence(5’-3’)	PCR or LDR length
rs3747823 G/A	rs3747823-up	CAAAGCAAGGCAAATGTGAA	159
	rs3747823-low	ATGGCTGGACATGGAAAAGT	
	rs3747823_modify	P-TTCTTCATCTTCAAAATGAGATTTCTTTTTTT TTTTTTTTTTTTTTTTTGCATCACTCA-FAM	
	rs3747823-A	TTTTTTTTTTTTTTTTTTCTCTGTCTGAGTCTCT GT	150
	rs3747823-G	TTTTTTTTTTTTTTTTTTTTCTCTGTCTGAGTCT CTGC	152
rs7020782 A/C	rs7020782-up	GCACTTCGCATGTGAGAAAA	186
	rs7020782-low	GAGCTCAGCACCTGCACATA	
	rs7020782-modify	P-AAGCATTCTCCACAGCCAGCTCTGGTTTTTT TTTTTTTTTTTTTTTTTTGCATCACTCA-FAM	
	rs7020782-A	TTTTTTTTTTTTTTTTTTTTTTTTTGCGGTCGCT GCTGGAGCAATTGAGAT	165
	rs7020782-C	TTTTTTTTTTTTTTTTTTTTTTTTTTTGCGGTCG CTGCTGGAGCAATTGAGAG	167
rs13290387 G/C	rs13290387-up	GGGAGAATCCTGGTGCAGTA	249
	rs13290387-low	AAATGGATGAGCGCTTGACT	
	rs13290387-modify	P-AAAGACCCATAAAAATAATAAAACCTTTTTT TTTTTTTTTTTTTTTTTTGCATCACTCA-FAM	
	rs13290387-C	TTTTTTTTTTTTTTTTTTTTTTTTTTTTTTTGTTAC TCAAATGAAATGATACATGGG	170
	rs13290387-G	TTTTTTTTTTTTTTTTTTTTTTTTTTTTTTTTTGTT ACTCAAATGAAATGATACATGGC	172

PCR, polymerase chain reaction; LDR, ligase detection reaction
